# Prospective cohort study protocol to evaluate the validity and reliability of the Quality of Trauma Care Patient-Reported Experience Measure (QTAC-PREM)

**DOI:** 10.1186/1472-6963-13-98

**Published:** 2013-03-14

**Authors:** Niklas Bobrovitz, Maria Santana, Theresa Kline, John Kortbeek, Henry T Stelfox

**Affiliations:** 1 Department of Community Health Sciences, University of Calgary, Calgary, AB T2N 4Z6, Canada; 2 Department of Medicine, University of Calgary, Calgary, AB T2N 4Z6, Canada; 3 Institute of Public Health, University of Calgary, Calgary, AB, T2N 4Z6, Canada; 4 W21C Research and Innovation Centre, University of Calgary, Calgary, AB, T2N 4Z6, Canada; 5 Department of Critical Care Medicine, University of Calgary, Calgary, AB, T2N 4Z6, Canada; 6 Department of Psychology, University of Calgary, Calgary, AB, T2N 4Z6, Canada; 7 Department of Surgery, University of Calgary, Calgary, AB, T2N 4Z6, Canada; 8 Teaching Research & Wellness Building, University of Calgary, 3280 Hospital Drive NW, Calgary, AB, T2N 4Z6, Canada

**Keywords:** Measure, Survey, Injury, Experience, Satisfaction, Patient experience, Patient-centered, Patient-reported

## Abstract

**Background:**

Patient-centeredness is a key component of health care quality. However, patient-centered measures of quality have not been developed in injury care. In response to this challenge, we developed the Quality of Trauma Adult Care Patient-Reported Experience Measure (QTAC-PREM) to measure injured patient experiences with trauma care and pilot-tested the instrument at a single Level 1 trauma centre. The objective of this study is to test the reliability, validity, and feasibility of the QTAC-PREM in multiple Canadian trauma centers and to refine the measure based on the results.

**Methods/design:**

This will be a prospective cohort study of consecutive adult (age ≥ 18 years) patients discharged from three trauma centres in Alberta, Canada with a primary diagnosis of injury. The target sample size is 400 participants to ensure precision for evaluating test-retest reliability. We will assess the psychometric properties of the measure (test-retest reliability, construct validity, internal consistency) and whether these properties vary by patient characteristics. We will also evaluate the predictive validity, convergent validity, and discriminant validity of the measure against other established tools (HCAHPS).

**Discussion:**

A reliable and valid measure of patient reported experiences with injury care may be a valuable tool to evaluate quality of care and guide improvement efforts.

## Background

### The problem: burden of injury and the quality of care

Globally, injuries affect 700 million people each year, including 30 million North Americans [1,2]. Although healthcare systems provide patients with vital treatment for this major cause of morbidity and death, assessments of the quality of injury care demonstrate that care often fails to meet established standards [3]. Studies show up to half of all critically injured patients do not receive recommended care [4], adverse events are common [5], and injury care may not meet the needs of certain patients. For example, patients with traumatic brain injuries, as well as their families, report deficits in information provided by health care professionals [6].

### Measurement is necessary for improvement

In order to improve care, valid and reliable information on the quality of care is necessary. Healthcare professionals and organizations can use quality measurement tools to identify discrete problem areas, to aid in tailoring interventions to correct care issues, and to track subsequent improvements. Healthcare regulators may use quality information to develop quality-monitoring processes, to target inspections, and to document gaps in optimal care.

### Measuring patient experiences: a key element to improving care

To date, quality measurement and improvement efforts in injury care have primarily focused on clinical processes and outcomes of care such as risk-adjusted mortality [7]. However, ‘quality’ in healthcare is composed of more than clinical processes and outcomes. Quality of care has been defined by The Institute of Medicine (IOM) as “the degree to which health services for individuals and populations increase the likelihood of desired health outcomes and are consistent with current professional knowledge” [8]. The IOM emphasizes that ‘desired outcomes’ are a composite of patient and clinical goals such that care is patient-centered: respectful of and responsive to individual patient preferences, needs, and values. Therefore, measuring patient care experiences is a central element for assessing and improving the quality of injury care.

Capturing patient perspectives is often achieved with self-administered survey based measures. This approach allows patients to complete the measure when it is convenient for them to do so, and provides respondents time to think about the questions they are asked. Survey based measures are also relatively easy to administer; data can be obtained from large samples relatively quickly, and can be inexpensive.

Survey items measuring patient experiences or satisfaction with care usually consist of a patient-defined expectation or standard of care and an evaluation of the degree to which the expectation or standard was met in the patient’s experience [9]. A key challenge to developing a valid instrument is to identify aspects of care that are relevant to patients’ perceptions of the quality of care. Quality is not composed of a single aspect of care. Valid measures contain multiple care domains that serve as constructs in the patients’ conception of quality. These components of care have been shown to vary across different patient populations [10].

### A gap in trauma quality of care measurement and improvement

Patient-centered measures have been developed in select areas of healthcare. For example, the Consumer Assessment of Healthcare Providers and Systems (CAHPS) program (of the U.S. Agency for Healthcare Research and Quality) has developed valid and reliable measures of patient experiences with ambulatory (primary care, home care) and in-hospital medical-surgical (excluding trauma) care [11]. These consumer-assessments of care measures have been used extensively in the United States and have been successfully used to identify deficits in care delivery and support quality improvement [12-15]. For example, a study of eight collaborative medical groups focused on patient-centered care in Minnesota successfully used a modified CAHPS survey measure to identify opportunities for care improvement, develop quality improvement interventions, and produce measurable improvements in patient experience [12].

Despite the central importance of patient perspectives of care and the demonstrable value of patient-centered measures, to date there has been limited progress in incorporating patient perspectives into quality measurement in injury care. Only a few instruments have been developed for assessing patients’ experiences or satisfaction with injury services. Many of the studies measuring patient perspectives of quality in injury care have used non-validated measures to assess patient perspectives of specific interventions or injury treatments [16-18]. A small number of reliable and valid measures exist, but were developed for specific patient populations (e.g. head injured patients) or specific injury care services (e.g. rehabilitation services) [17-19]. For example, surveys of traumatic brain injured patients and their families have highlighted deficits in care related to information and follow-up [6,20]. While this is an important start to measuring patient experiences with injury care, assessments of the broader population of injured patients are needed. Currently, there are no published measures designed to capture the overall healthcare experiences of patients with major injuries and as a result, it is not possible to comprehensively evaluate the quality of care provided to injured patients they receive.

### Measure development

To address this gap in trauma quality improvement we developed the Quality of Trauma Adult Care Patient-Reported Experience Measure (QTAC-PREM) using a comprehensive literature review and focus groups with key trauma stakeholders [21]. The measure is comprised of two components, one to evaluate acute care (hospital) and the other post-acute care (discharge, follow-up). The measure is designed to be completed by patients and evaluate their experiences with injury care. However, because some injured patients (e.g. severe traumatic brain injured patients) cannot complete a survey, we also developed a family member version to allow for proxy measurement of patient experience [22].

Pilot evaluation at a single trauma centre showed the measure to be feasible to implement (81% overall response rate) and provided preliminary evidence of content and construct validity. The pilot study highlighted several areas of care for potential improvement including: caregivers dealing with patient concerns, information about the effect of injuries on the patients’ lives, information about the recovery process, treatment of agitation and anxiety, consideration of personal hygiene and patients’ emotional needs, and inconsistency of information. Additional areas of post-hospital care highlighted for improvement were: information about discharge and home care, and family physicians not receiving hospital discharge information. For the most part, our findings were consistent with studies of patient and family experiences with general hospital care [23], intensive care unit services [24], and injury care [6,25].

The next step in this research is to evaluate the psychometric properties of the measure in multiple trauma centres. External validation, through a multi-centre study, is needed to ensure the reliability, content validity and construct validity of the measure hold across different settings and different participants. This would increase the generalizability and comparability of the results and thereby increase the value of the measure as a quality improvement tool. A larger validation sample also provides the opportunity to create a short form version of the measure to decrease respondent burden and potentially increase response rates in the future.

The primary objectives of this study are:

**Objective 1:** To test the psychometric properties (test-retest reliability, construct validity, internal consistency, predictive criterion validity) and feasibility of the Quality of Trauma Adult Care Patient-Reported Experience Measure (QTAC-PREM) in multiple Canadian trauma centers.

**Objective 2:** To assess whether the construct validity of items varies by patient clinical or demographic characteristics.

**Objective 3:** To refine the measure based on results of the multi-center study to improve efficiency and usability.

The secondary objectives of this study are:

**Objective 4:** To assess the correlation between patient survey measure responses and patient family member’s survey measure responses.

**Objective 5:** To assess the convergent validity of the measure with the Hospital Consumer Assessment of Healthcare Providers and Systems (HCAHPS) survey measure.

**Objective 6:** To assess the divergent validity of the measure with the Hospital Consumer Assessment of Healthcare Providers and Systems (HCAHPS) survey measure.

## Methods/design

### Design

This will be a prospective cohort study of consecutive adult (age ≥ 18 years) patients admitted to hospital due to injury. We will sample all patients admitted with a primary injury diagnosis as we want to obtain a comprehensive picture of the quality of injury care. We define injury as “the physical damage that results when a human body is suddenly subjected to energy in amounts that exceed the threshold of physiological tolerance” [26] resulting in admission to hospital.

### Recruitment and data collection

#### Part 1: in-hospital acute care measure

We propose to implement the survey measure at three trauma centres in the province of Alberta. Research coordinators will conduct daily screening of patients admitted for traumatic injuries in conjunction with trauma program coordinators and charge nurses. This study has received approval from the Conjoint Health Research Ethics Board at the University of Calgary (primary site ethics I.D.: E-24364).

Eligible participants will be those admitted to hospital with a primary injury diagnosis. Patients that are unable to understand and consent to study procedures will not be eligible to complete the survey measure themselves. Patient capacity to provide informed consent will be determined using a modified Aid to Capacity Evaluation form (ACE) [27]. If a patient is unable to provide informed consent (e.g. head injury), a family member will be invited to participate by completing the surrogate consent form and survey measure as a surrogate. Family members of patients admitted due to injury will be eligible if they visit their injured relative at least one time in hospital. We will exclude family members of patients who die during their hospital stay.

**Figure 1:**
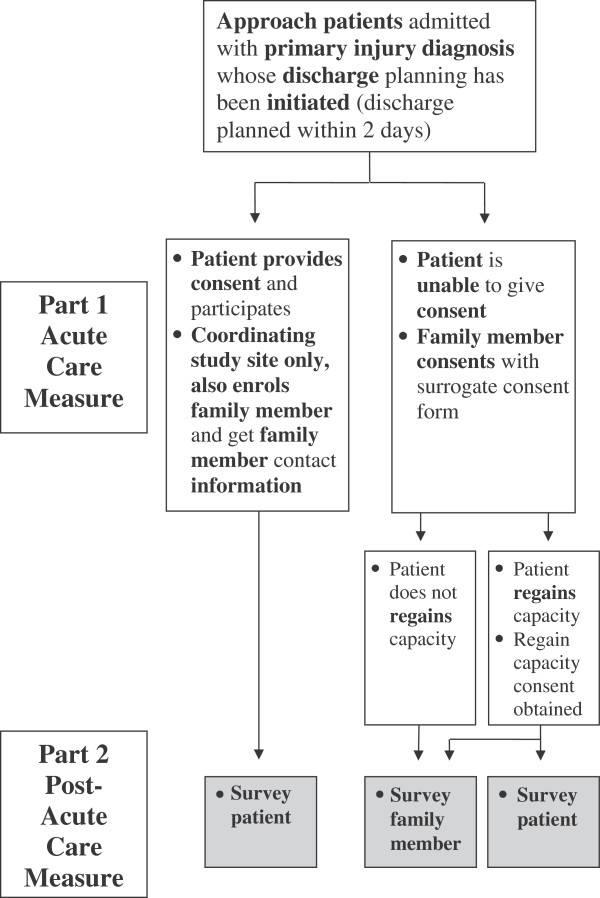
Recruitment flow chart.

Participant recruitment and study flow is outlined in Figure 1 and will be conducted according to the following procedure:

1. Research coordinators will conduct daily screening (Monday through Friday) of patients admitted due to injury for whom discharge planning has been initiated (planned discharge within 2 days).

2. Research coordinators will approach eligible patients and family members prior to discharge, describe the study, and invite them to participate. The research coordinators will use the Aid to Capacity Evaluation form [27] to evaluate the capacity of patients for whom there is concern about their ability to provide informed consent. If a patient is unable to provide informed consent (e.g. severely head-injured), one of their family members will be offered participation. If a patient is able to provide informed consent, but would prefer that a family member participant (e.g. too tired, nauseated etc.) one of their family members will be offered participation.

3. Research coordinators will administer one in-hospital acute care survey measure per consenting patient/family. To evaluate the concordance of patient and proxy/family responses a second survey measure will be offered to eligible family members of participating patients (administration of second surveys restricted to coordinating study site).

4. To assess reliability, consecutive participants will be approached to re-complete the survey measure within 24–48 hours of initial survey administration until the required sample is reached (n = 75; ~25 per site).

5. Once we have achieved the sample for the reliability assessment at the coordinating study site (~n = 25) we will initiate one of the site-specific secondary objectives: to assess convergent and divergent validity with the HCAHPS measure. Consecutive patients will be approached at the coordinating study site to complete the HCAHPS measure concurrently with the QTAC-PREM. We aim to administer the HCAHPS to approximately 50 participants.

6. The completed in-hospital acute care survey measure will be sealed in an envelope and numbered with a unique letter/number code.

#### Part 2: after-hospital post-acute care measure

Patients/families that complete the in-hospital acute care survey measure will be offered to participate in the post-acute care survey. Consenting patients/families will be contacted by telephone 8–12 weeks post-discharge by an experienced telephone interviewer (NB).

7. A telephone script will be used to:

a) Remind patients and family members of the purpose of the study.

b) Re-evaluate patient capacity to provide informed consent. Family members of patients previously unable to provide consent in-hospital (e.g. head injured) will be asked about the condition of their recovering relative. If deemed appropriate (i.e. capacity potentially regained), patients will be contacted to assess their capacity to consent (using the modified version of the Aid to Capacity Evaluation tool [27]) and to discuss their willingness to continue participation in the study.

8. The interviewer will record participant answers on a paper copy of the post-acute care measure. Completed survey measures will be linked to participant’s acute care survey measure using unique letter/number codes. To assess test-retest reliability of the post-acute care measure it will be re-administered via telephone interview to consecutive participants 7–10 days after the initial completion until the required sample size is reached (n = 75).

### QTAC-PREM

Our measure has undergone multiple revisions based on our pilot-test and cognitive interviews with injured patients and their family members. The acute care measure (Additional file 1) consists of seven domains: *communication and information, transfers and patient transport, pain management, comfort, interpersonal care, safety, and equality*. This measure includes 35 items: 7 demographic, 1 health status, 4 open-ended, and 23 close ended. The post-acute care measure (Additional file 2) consists of six domains: *communication and information, pain management, access, interpersonal care, coordinated care, and safety*. This measure includes 22 items: 3 open-ended and 19 close-ended. This measure uses screening questions to determine which set of items participants will be asked. Participants only answer questions about care they have received.

### Sample size

Our sample size is based on ensuring precision for test-retest reliability. Previous validation studies on similar populations found survey item correlation estimates to be in the range of 0.65 to 0.85 [28]. A test-retest sample size of 75 with an intra-class correlation (ICC) of 0.75 corresponds to 95% CI width of +/- 0.10. We will conduct re-tests on 25% of participants (both for the acute care and post-acute care survey measures), estimate that 25% of participants will decline requests for re-tests, and therefore, estimate the need to recruit approximately 400 participants.

### Data analysis

#### *Objective 1*

i) We will assess test-retest reliability by calculating Pearson correlation coefficients at the level of individual items and for each composite domain.

ii) Confirmatory factor analysis will be performed to assess construct validity by determining if items load adequately (factor loadings >0.3) [29,30] onto a domain category. To perform this analysis individual binary and categorical item scores will be transformed to linear values on the standard normal distribution.

iii) Corrected item-to-total correlation coefficients will be calculated between each item and both the domain and global item score to assess construct validity.

iv) Internal consistency analyses will be conducted on the factors resulting from confirmatory factor analysis. We will determine the consistency of items within each factor by examining Cronbach’s α coefficients for each domain and across the entire set of items. To perform this analysis, item scores will be transformed to linear values on the standard normal distribution.

v) We will examine predictive criterion validity of both the acute care measure and post-acute care measure in two ways:

a) We will use ordinal regression to determine if the individual item scores are related to the scores of the global rating items. Individual items will be used as explanatory variables and the 0–10 global rating scales will be treated as ordinal outcome data.

b) We will use ordinal regression to determine if the scores on the composite subscales (domains) are related to the scores of the global rating items. Individual item scores will be transformed to values on the standard normal distribution. A mean of the item scores will be calculated for each subscale and used as the summary subscale value. Missing values will be replaced by substitution of the mean value (sensitivity analysis will be performed using complete cases). The 0–10 global rating scales will be treated as ordinal outcome data.

vi) We will examine predictive criterion validity using two additional approaches for the acute care measure:

a) We will use ordinal regression to determine if the injury care global rating score on the acute care measure is related to the injury care global rating score on the post-acute care measure.

b) We will use multiple logistic regression to determine if subscale scores or the global rating scores on the acute care measure predict whether patients register formal hospital complaints or compliments. Subscale scores will be calculated by taking a linear mean of item scores after converting them to values on the standard normal distribution. The injury care global rating score will be treated as ordinal data. Registration of a formal complaint or compliment will be treated as a binary outcome (yes/no).

vii) We will examine the survey response rate (percentage of respondents who completed the measure among all those approached to participate in the study) to determine if implementing the measure is feasible in multiple trauma centres.

#### *Objective 2*

We will re-run the factor analysis using subgroup categories to determine if the factor structure varies by key patient characteristics. Differences will be determined by examining the pattern of loading/non-loading (>0.3 vs <0.3 respectively) [29,30] in each group. Given the sample size requirements for factor analysis (about 10 participants per item is expected) [29,30] it may only be possible to examine select characteristics. We will first analyze sex (male vs. female) and injury severity score (minor injury ISS <15 vs. major injury ISS >15). Secondary analysis will be performed on the following demographic variables if sample size permits: ethnicity, age, length of hospital stay, education level, and need for surgery or intensive care during hospital stay [31].

#### *Objective 3*

Item reduction will be based on principal axis factor analysis assessments of internal consistency. Redundant items (item-scale Cronbach’s α >0.8) [32] and items that do not adequately load on a factor (factor loadings <0.3 in principal axis factor analysis) [29,30] will be eliminated.

Item scores will be transformed to linear values on the standard normal distribution for objectives 4-6 [33].

#### *Objective 4*

We will calculate intraclass correlation coefficients to determine the degree of concordance between patient survey measure responses and their family member’s survey measure responses.

#### *Objective 5*

Assessments of convergent validity will be based on intraclass correlation coefficients calculated between domains on the patient experiences measure and the HCAHPS survey measure. A coefficient of 0.9 will be the cut-off for collinearity [34].

#### *Objective 6*

Assessments of divergent validity will be based on intraclass correlation coefficients calculated between domains on the patient experiences measure and the HCAHPS survey measure. A coefficient of 0.4 will be the cut-off for collinearity [35].

The open-ended responses on the survey measure will be qualitatively analyzed. We will use a mixed inductive/deductive thematic analysis to identify major themes of patient experiences with injury care. This will include both the data-drive inductive approach outlined by Boyatzis [36] and the use of an a priori coding framework in the deductive approach outlined by Crabtree and Miller [37].

To assess non-response bias we will compare respondents’ characteristics to the average characteristics of the injury population admitted during the same time frame in each institution. Differences will be assessed using Students t-test and Mann–Whitney U comparisons.

## Discussion

### Implications

We believe a reliable and valid measure will be a valuable tool to evaluate patient experiences with injury care and guide quality improvement efforts.

If the measure is found to have desirable levels of validity and reliability, we will eliminate redundant items and begin to plan a multi-center study to assess patient experiences with injury care. Additional inquiry would be focused on two areas of investigation. First, to study the relationship between patient experiences of injury care and important patient outcomes including patient quality of life post-injury, hospital readmission after discharge (for an issue related to the initial injury), and primary care utilization after discharge. Second, to examine the relationship between patient reported experiences of care and clinical measures of care quality (Santana MJ, Stelfox HT, Straus S: Development and evaluation of the evidence-informed quality indicators for adult injury care, forthcoming).

If the measure is found not to have desirable levels of validity and reliability we will identify specific deficits and pursue appropriate revisions. If issues with validity are identified we will revise the measure’s content and items using small focus groups with injury patients and family members to ensure we are addressing priority aspects of injury care for patients. To address issues of reliability we will conduct cognitive interviews with patients to improve the clarity of items and consistency of item interpretation.

### Potential limitations and challenges

There are a few potential limitations to the proposed study. First, there is the possibility of response bias. We will assess non-response bias by comparing respondents’ characteristics to the average characteristics of the injury population admitted to each institution during the same time frame as data collection. A second limitation is that we will not sample family members of patients that died during their hospital stay. As a result, a modified QTAC-PREM will need subsequent evaluation in this population. A third limitation is the possibility of social desirability bias, a common threat to measure validity [38,39]. It is possible that completion of the measure in-hospital may lead to increased social desirability bias compared to mail-out survey measures. However, this issue must be balanced against the problem of recall bias, as well as feasibility. We selected in-hospital administration to reduce recall bias and to improve response rate; studies show response rates are often lower for mail-out surveys than on-site administration [40]. However, an additional limitation with administering surveys prior to discharge is that we may miss key aspect of care during the discharge process. To mitigate this we have included items about the discharge process in the post-acute care measure. A fourth limitation is our inability to quantify the agreement between surrogate responses and those of patients with severe traumatic brain injuries. This is an unavoidable limitation given the involvement of patients who do not have capacity to provide informed consent. Although we cannot be sure how accurate surrogate responses are of the perspectives of patients with severe traumatic brain injury, we may obtain an estimate by examining agreement between responses of patients with minor traumatic brain injury and those of one of their family members.

We foresee at least one key challenge to this study: the possibility of slow recruitment. This will be partially dependent on the volume of trauma patients passing through each centre. However, to ensure efficient recruitment of admitted trauma patients we have developed a detailed recruitment package to be used by research coordinators at each site. This package includes dialogue excerpts that were used during a previous pilot-testing phase that resulted in over 85% response rates in-hospital. In addition, one of the team members (NB) will conduct site visits to address recruitment concerns of research coordinators and ensure consistency in the recruitment strategy across the recruiting sites.

## Conclusion

We propose to conduct a prospective cohort study to evaluate the reliability and validity of the Quality of Trauma Adult Care Patient Reported Experience Measure (QTAC-PREM). This is the first survey measure designed to capture patient experiences with acute and post-acute injury care. If the measure is found to have acceptable levels of validity and reliability it may be a valuable tool to evaluate patient experiences with injury care and guide quality improvement efforts.

## Competing interests

The project is supported by a Partnerships in Health System Improvement Grant (PHE-238551) from the Canadian Institutes of Health Research and Alberta Innovates. Nik Bobrovitz is supported by a Health Quality Council of Alberta Studentship and an Alberta Innovates Health Solutions Studentship. Dr. Stelfox is supported by a New Investigator Award from the Canadian Institutes of Health Research and a Population Health Investigator Award from Alberta Innovates. Funding sources will have no role in the design of this study and will have no role in the conduct or reporting of this study. We are unaware of any conflicts of interest. None of the authors have financial or professional conflicts of interest that would influence the conduct or reporting of this study.

## Authors’ contributions

NB, MS, TK, JK, and HTS contributed to the design of this study. NB and HTS drafted the manuscript. NB, MS, TK, JK, and HTS contributed critical revisions of this manuscript and gave final approval of this version of the manuscript for publication. NB will acquire the data. NB, MS, TK, JK, and HTS will be involved in the analysis and interpretation of the data. All authors read and approved the final manuscript.

## Pre-publication history

The pre-publication history for this paper can be accessed here:

http://www.biomedcentral.com/1472-6963/13/98/prepub

## Supplementary Material

Additional file 1 **Quality of Trauma Care Patient-Reported Experience Measure (QTAC-PREM).** Part 1: Acute Care, Patient Survey. Click here for file

Additional file 2 **Quality of Trauma Care Patient-Reported Experience Measure (QTAC-PREM).** Part 2: Post-Acute Care, Patient Survey. Click here for file
