# Supplementary material for: Prospective cohort study protocol to evaluate the validity and reliability of the Quality of Trauma Care Patient-Reported Experience Measure (QTAC-PREM)
Source: BMC Health Serv Res. 2013 Mar 14;13:98. doi: 10.1186/1472-6963-13-98 (PMC3623625; doi:10.1186/1472-6963-13-98)
Supplement: Additional file 1 — Quality of Trauma Care Patient-Reported Experience Measure (QTAC-PREM). Part 1: Acute Care, Patient Survey. [file 1472-6963-13-98-S1.doc]

**Quality of Trauma Care Patient-Reported Experience Measure (QTAC-PREM)**

**Part 1: Acute Care, Patient Survey**

| - **We are conducting a study on injury care and want to know about your experiences. We are interested in all of your thoughts, especially on what can be improved. We understand this is a difficult time for you but we would appreciate you taking the time to tell us about your care.** - **All information is confidential (your healthcare providers will not see this information).** - **Your responses will be used to improve care.** |
| --- |

**Demographics**

**1.** **What is your sex?**

Female

Male

**2.** **What is your age? (in years)**

**3. Where do you live (city/town, province)?**

**4. How were you injured?**

Car Pedestrian-Vehicle

Fall Bicycle Burn

Assault Attempted Suicide

Other (please specify):

**5.** **What is the highest level of education you**

**have completed?**

8th grade or less

Some high school, but did not graduate

High school graduate

Some college/university, did not graduate

College diploma/certificate

4 year university degree

More than 4 year university degree

**6. What is your ethnicity?**

Caucasian

Black or African Canadian

Asian

Aboriginal or First Nations

East Indian

Latin American

Other (please print):

**7. What language do you mainly speak at**

**home?**

English French Vietnamese

Spanish Chinese (Mandarin/Cantonese)

Other (please print):

**8. Since being injured, which of the following**

**options best describes your overall health**

**status?**

Excellent

Very good

Good

Fair

Poor

| - **Your care for this injury may have included: care in the emergency department, intensive care unit, trauma care unit, and rehabilitation in the hospital.** - **Think ONLY about the care you have received for this injury when answering the questions.** - **Answer all the questions by checking the response box to the left of your answer.** - **Please CHECK ONLY ONE response for each question. There is room for general comments at the end of the survey.** |
| --- |

**During your care for this injury…**

**Communication and Information**

**9**. **How often did your healthcare providers**

**(e.g. doctors, nurses, therapists, etc) explain**

**things in a way you could understand?**

Never

Sometimes

Usually

Always

**10. Did your healthcare providers clearly**

**explain all your injuries to you in a way**

**you could understand?**

Yes

No

Not able to answer

**11. Did your healthcare providers discuss how**

**the injuries might affect you after you**

**leave the hospital?**

Yes

No

Not able to answer

**12. Did your healthcare providers provide**

**instructions on how you should care for**

**your injuries after you leave the hospital?**

Yes

No

Not able to answer

**13. Did your healthcare providers discuss how**

**long it might take you to recover from**

**your injuries?**

Yes

No

Not able to answer

**14. How often was the information you**

**received from your various healthcare**

**providers consistent?**

Never

Sometimes

Usually

Always

**Transfers and Patient Transport**

**Injured patients are transported around the hospital and sometimes between hospitals. Patients can be moved from the emergency department or intensive care unit to a general hospital unit and to different areas of the hospital to receive tests, scans, and surgeries.**

**15.** **During your transfers did the hospital**

**staff or healthcare providers clearly**

**explain where you were being transferred**

**to?**

Yes

No

I don’t know

**During your care for this injury…**

**16.** **During your transfers, how often were you**

**kept comfortable?**

Never

Sometimes

Usually

Always

**17. When you arrived to a new hospital unit**

**did a healthcare provider explain where**

**important landmarks were in the unit?**

**(e.g. call button, bathroom,**

**nurse’s station, water/ice machine)**

Yes

No

I don’t know

**Pain Management**

**18. How often was your pain well controlled?**

Never

Sometimes

Usually

Always

Not Applicable- did not have pain

**19. How often did the healthcare providers**

**do everything they could to help you with**

**your pain?**

Never

Sometimes

Usually

Always

Not Applicable- did not have pain

**Comfort**

**20. How often did the healthcare providers**

**do everything they could to help you with**

**your difficulty breathing?**

Never

Sometimes

Usually

Always

Not Applicable- no difficulty breathing

**21. How often did the healthcare providers**

**do everything they could to help you with**

**your agitation or irritability?**

Never

Sometimes

Usually

Always

Not Applicable- no agitation or irritability

**22. When the healthcare providers rolled you,**

**turned you over in bed, or helped you**

**get out of bed and move around, how often**

**did they do it carefully?**

Never

Sometimes

Usually

Always

Not Applicable- did not need help moving

**23. How often did your nurses or other**

**hospital staff help you maintain your**

**personal hygiene?**

Never

Sometimes

Usually

Always

Not Applicable- did not need/want help

**During your care for this injury…**

**Interpersonal Care**

**24. When meeting a new healthcare provider**

**for the first time how often did they**

**introduce themselves and clearly explain**

**their role in your care?**

Never

Sometimes

Usually

Always

**25. When you expressed concerns or**

**frustrations about your care how often did**

**your healthcare providers take action to**

**deal with them?**

Never

Sometimes

Usually

Always

Not Applicable- had no concerns

**26. Did a healthcare staff member**

**(e.g. psychologist, social worker, nurse) offer to**

**speak with you about your emotional**

**needs?**

Yes

No

**27. How often was your dignity considered by**

**the healthcare providers?**

Never

Sometimes

Usually

Always

**Safety**

**28. How often did you experience care that**

**was unsafe? (e.g. medication/treatment errors,**

**complications)**

Never

Sometimes

Usually

Always

**Equality**

**29. How often were your cultural, religious, or**

**spiritual preferences respected by the**

**healthcare staff and religious or spiritual**

**staff?**

Never

Sometimes

Usually

Always

Not Applicable

**30. How often were you treated unfairly**

**because of your age, ethnicity, gender, or**

**personal characteristics?**

Never

Sometimes

Usually

Always

**During your care for this injury…**

**Overall Care**

**31. Please provide an overall rating of the care**

**you have received for this injury.**

**0** Worst Injury Care Possible

**1**

**2**

**3**

**4**

**5**

**6**

**7**

**8**

**9**

**10** Best Injury Care Possible

**32. What was the best aspect of the care you**

**received?**

**33. What was the worst aspect of the care you**

**received?**

**34. Describe any care you received that was**

**unsafe.**

**(e.g., medication/treatment errors, complications)**

**35. What can we do to improve care for injury**

**patients?**

**When you have finished the survey, please fold it, put it in the envelope given to you, and return it to the research assistant. If you have any questions please feel free to ask them of the research assistant. Thank you for sharing your experience with us. This will help us improve care.**
