# Supplementary material for: Prospective cohort study protocol to evaluate the validity and reliability of the Quality of Trauma Care Patient-Reported Experience Measure (QTAC-PREM)
Source: BMC Health Serv Res. 2013 Mar 14;13:98. doi: 10.1186/1472-6963-13-98 (PMC3623625; doi:10.1186/1472-6963-13-98)
Supplement: Additional file 2 — Quality of Trauma Care Patient-Reported Experience Measure (QTAC-PREM). Part 2: Post-Acute Care, Patient Survey. [file 1472-6963-13-98-S2.doc]

**Quality of Trauma Care Patient-Reported Experience Measure (QTAC-PREM)**

**Part 2: Post-Acute Care, Patient** Survey

| - **This survey contains questions about your discharge from the hospital and about the follow-up care you have received since leaving the hospital.** - **I will ask you questions and read the response options to you. Please tell me the response option that best reflects your experience.** - **This survey will take about ten minutes to complete.** - **There are twenty-two questions.** |
| --- |

| **Text Box 1- Read** |
| --- |
| The first question is…. |

1. **Where did you go after being discharged from [*hospital name]* ?**

Home 🡪 go to text box **3**

A friend or family member’s home 🡪 go to

text box **3**

Another hospital 🡪 go to text box **2**

An in-patient rehabilitation facility 🡪 go to

text box **2**

A long term care facility 🡪 go to question **6**

| **Text Box 2- Read** |
| --- |
| When answering questions in this survey, think about the final hospital or healthcare facility that you were discharged from, not [*hospital name*]. |

| **Text Box 3- Read** |
| --- |
| I am now going to ask you ten questions and you can answer using the following response scale: yes, no, or not applicable. |

**Discharge Process**

*Communication and Information*

1. **Before leaving the hospital, did your hospital healthcare providers give you written instructions on how to care for your injuries after being discharged?**

Yes 🡪 go to question **3**

No 🡪 go to question **4**

Not Applicable🡪 go to question **4**

1. **Did the written instructions you received provide you with enough information to help you care for your injuries after being discharged?**

Yes

No

Not Applicable

*Pain Management*

1. **After being discharged from the hospital,**

**did you have enough pain medication to**

**control your pain well?**

Yes

No

Not Applicable

*Access*

1. **After being discharged from the hospital,**

**did you get the all of the support services that**

**you wanted, for example, home care, social**

**work, or counselling?**

Yes

No

Not Applicable

**Services Accessed**

1. **Since being discharged from the hospital, have you attended an appointment to follow-up about your injuries with…**
2. A trauma doctor, surgeon, or specialist?

Yes

No

Not Applicable

1. A family doctor or general practitioner?

Yes

No

Not Applicable

1. A physio, rehabilitation or occupational therapist?

Yes

No

Not Applicable

1. **Are you scheduled for an appointment to follow-up about your injuries with…**
2. A trauma doctor, surgeon, or specialist?

Yes

No

Not Applicable

1. A family doctor or general practitioner?

Yes

No

Not Applicable

1. A physio, rehabilitation or occupational therapist?

Yes

No

Not Applicable

| **Text Box 4- Question Guide** |
| --- |
| Only ask about providers in question 8 if participant indicated YES to that provider in question 6 or 7. |

*Access*

1. **Did you have difficulty getting follow-up**

**appointments when you wanted them with…**

1. A trauma doctor, surgeon, or specialist?

Yes

No

Not Applicable

1. A family doctor or general practitioner?

Yes

No

Not Applicable

1. A physio, rehabilitation or occupational therapist?

Yes

No

Not Applicable

| **Text Box 5- Question Guide** |
| --- |
| If NO to 6a,b,c and 7a,b,c 🡪 go to question **17**.  If YES to 6a,b,c or 7a,b,c🡪 go to text box **6**. |

| **Text Box 6- Read** |
| --- |
| I’m now going to ask you about your follow-up appointments. These questions apply to all of the healthcare providers you have seen since being discharged from the hospital. |

*Communication and Information*

1. **During your follow-up appointments, did**

**your healthcare providers explain the next**

**steps in your recovery from injury for**

**example, activities you should or should not do,**

**necessary medications, tests and treatments,**

**or other follow-up appointments?**

Yes

No

Not Applicable

1. **During your follow-up appointments, did**

**your healthcare providers explain**

**approximately how long it would take you to**

**recover?**

Yes

No

Not Applicable

1. **During your follow-up appointments, did**

**you get all of the information that you**

**wanted from the healthcare providers?**

Yes

No

Not Applicable

| **Text Box 7- Read** |
| --- |
| The next four questions use a different response scale. It is a four point frequency scale. The options are as follows: one is never, two is sometimes, three is usually, and four is always. There is also the option of not applicable. |

*Communication and Information*

1. **During your follow-up appointments, how**

**often did your healthcare providers explain**

**things about your injuries in a way you could**

**understand?**

Never

Sometimes

Usually

Always

Not Applicable

*Interpersonal Care*

1. **During your follow-up appointments, when you expressed concerns or frustrations how often did your healthcare providers take action to deal with them?**

Never

Sometimes

Usually

Always

Not Applicable

| **Text Box 8- Question Guide** |
| --- |
| If YES to more than one option (a ,b ,c) in question 6🡪 go to question **14**.  If YES to only one option (a, b, c) in question 6🡪 go to question **15**. |

*Coordinated Care*

1. **After being discharged from the hospital, how often was the information you received from your different healthcare providers consistent?**

Never

Sometimes

Usually

Always

Not Applicable

*Safety*

1. **Since being discharged from the hospital, how often have you experienced healthcare that was unsafe?**

Never

Sometimes

Usually

Always

Not Applicable

| **Text Box 9- Read** |
| --- |
| The next questions use a scale from zero to ten. I will describe each one to you. |

| **Text Box 10- Question Guide** |
| --- |
| If YES to 6b🡪 go to question **16**.  If NO to 6b🡪 go to question **17**. |

1. **In your opinion, how much information about your hospital stay was communicated to your family physician or general practitioner on a scale of zero to ten, zero being no information and ten being all the information?**

**0** No Information

**1**

**2**

**3**

**4**

**5**

**6**

**7**

**8**

**9**

**10** All the Information

*Coordinated Care*

1. **How well were you guided through the recovery process by your healthcare providers after being discharged from the hospital on a scale of zero to ten, zero being poor guidance, ten being excellent guidance.**

**0** Poor Guidance

**1**

**2**

**3**

**4**

**5**

**6**

**7**

**8**

**9**

**10** Excellent Guidance

O*verall Care*

1. **On a scale of zero to ten, please provide**

**an overall rating of the follow-up care you have**

**received after being discharged from the**

**hospital, zero being the worst injury care**

**possible, ten being the best injury care**

**possible.**

0 Worst Injury Care Possible

**1**

**2**

**3**

**4**

**5**

**6**

**7**

**8**

**9**

**10** Best Injury Care Possible

| **Text Box 11- Read** |
| --- |
| There are only four questions left. |

1. **Which of the following options best describes your overall health status right now?**

Excellent

Very Good

Good

Fair

Poor

| **Text Box12- Read** |
| --- |
| The last 3 questions are open ended. I will read the questions to you and write down what you say**.** |

1. **What was the best aspect of the follow-up care you received after being discharged from**

**the hospital?**

1. **What was the worst aspect of the follow-up care you received after being discharged from the hospital?**

1. **What can we do to improve care for injury patients after they are discharged from hospital?**

| **Text Box 13- Read** |
| --- |
| That’s the end of the survey. Thank you so much for taking the time to speak with me. |
